# Interactive voice response - an automated follow-up technique for adolescents discharged from acute psychiatric inpatient care: a randomised controlled trial

**DOI:** 10.1186/2193-1801-2-146

**Published:** 2013-04-08

**Authors:** Björn Axel Johansson, Susanne Remvall, Rasmus Malgerud, Anna Lindgren, Claes Andersson

**Affiliations:** 1 Department of Health Sciences, Clinical Health Promotion Centre, Lund University, SE-205 02 Malmö, Sweden; 2 Psychiatry Region Skåne, Department of Child and Adolescent Psychiatry, Emergency Unit, Skåne University Hospital, SE-205 02 Malmö, Sweden; 3 Psychiatry Region Skåne, Department of Child and Adolescent Psychiatry, Open Care Unit, Helsingborg Hospital, SE-254 37 Helsingborg, Sweden; 4 Emergency Medicine, Department of Medicine, Skåne University Hospital, SE-205 02 Malmö, Sweden; 5 Department of Mathematical Statistics, Centre for Mathematical Sciences, Lund University, SE-223 62 Lund, Sweden; 6 Department of Criminology, Malmö University, SE-205 06 Malmö, Sweden

## Abstract

Follow-up methods must be easy for young people to handle. We examine Interactive Voice Response (IVR) as a method for collecting self-reported data. Sixty inpatients were recruited from a child and adolescent psychiatric emergency unit in Malmö, Sweden and called every second (N = 30) or every fourth (N = 30) day from discharge until first visit in outpatient care. A pre-recorded voice asked them to evaluate their current mood using their mobile phones. Average response rate was 91%, and 71% had a 100% response rate. Gender, age and length of inpatient treatment did not affect response rate, nor did randomisation. Boys estimated their current mood on average as 3.52 units higher than girls, CI = (2.65, 4.48). Automated IVR is a feasible method of collecting follow-up data among adolescents discharged from a psychiatric emergency unit.

## Introduction

It is estimated that about 15% of all adolescents suffer from a mental disorder that requires treatment (Blakemore 2008). For the most severely ill adolescents inpatient treatment is often required, at least for a short period of time. The trend goes towards very short hospital stays. For adolescents discharged from US community hospitals between 1990 and 2000 the average length of stay declined from 12 to 4 days (Case et al. 2007). The outcome of this development, including comparisons between different inpatient treatment periods, is insufficiently analysed. In order to improve treatment outcome, especially in outpatient care (Ginsburg 2006), it is essential to develop new follow-up methods that are easy to administer and offer high response rates.

### Interactive Voice Response

Interactive Voice Response (IVR) is an automated telephone system in which a central computer is programmed to administer incoming calls or to dial designated phone numbers. Subjects respond to questions by pressing a number on the telephone keypad. The method is flexible and has high validity, and real time assessment with IVR has showed a higher validity compared to delayed assessment methods. IVR has been used both as a follow-up technique and for interventions in different settings (Corkrey & Parkinson 2002; Stritzke et al. 2005; Andersson et al. 2007; Heron & Smyth 2010).

### IVR use among adolescents with psychiatric syndromes in psychiatric or paediatric care

We have identified three previous studies using IVR to follow-up adolescents with psychiatric syndromes treated in psychiatric or paediatric care. Different follow-up frequencies have been used, resulting in differences in response rates. In all of these studies, subjects have been asked to dial the IVR system to perform follow-ups.

Brodey et al. (Brodey et al. 2005) included 108 adolescents treated as inpatients for substance-related disorders. A 154 items questionnaire was administered four times during a 10–12 day period, using either a clinical interview, self-administered internet or IVR interviews. Eighty-four percent accepted to participate, 51% were male, and in total 88% completed all four assessments. The patients were satisfied with the IVR method.

In two studies IVR has been used to follow-up adolescents treated as outpatients. Kaminer et al. (Kaminer et al. 2006) included 26 adolescents treated as outpatients for substance-related disorders. The patients called the IVR system for 14 successive days to answer 14 questions on alcohol and drug use. Eighty-four percent accepted to participate, 65% were male, and in total 72% of the assessments were completed. Blackstone et al. (Blackstone et al. 2009) included 95 psychologically traumatised adolescents treated at a paediatric emergency unit. The subjects were randomised to call an IVR system and respond to 12 questions monthly, bi-weekly or weekly for 8 weeks after discharge. Seventy-three percent accepted to participate, 63% were male. In total, 14% responded to all follow-ups (monthly group 12%; bi-weekly group 9%; weekly group 20%).

These studies could be categorised as frequent (Kaminer et al. 2006) and non-frequent (Blackstone et al. 2009) IVR studies. To further evaluate the feasibility of IVR to follow-up adolescents with psychiatric disorders, we randomised adolescents discharged from emergency inpatient care to be followed-up frequently (every second day) or non-frequently (every fourth day) in order to evaluate differences in response rates when assessing their current mood during a maximum of 31 days after discharge. To our knowledge this is the first study where adolescents discharged from a psychiatric emergency ward are randomised to be contacted by an automated IVR system.

### The emergency unit

The emergency unit at the Department of Child & Adolescent Psychiatry in Malmö has 11 beds. All patients are admitted with a parent (Londino et al. 2003). About 200 patients are treated annually. Sixty percent of them are girls. Mean age is 16.5 years. The most common diagnosis is depression. Ninety-five percent of the patients are discharged to outpatient care within 6 to 7 days of admission.

## Aim

The primary aim of this study was to investigate whether automated IVR is a feasible method of collecting self-reported data on current mood among adolescents discharged from a child and adolescent psychiatric emergency unit, and secondly to evaluate differences in response-rate between groups with different follow-up frequencies.

## Methods

### Recruitment

Consecutive adolescents were recruited between December 2008 and November 2009. All patients had been subject to inpatient treatment and were asked to participate on the day of discharge. All patients were given a short written instruction about the follow-up procedure.

### Randomisation

Each subject was randomised to be contacted by the IVR system either every second day (N = 30) or every fourth day (N = 30). These frequencies were considered reasonable as the first visit in outpatient care usually occurs within three weeks of discharge. Before the study was initiated, 30 green and 30 red marbles were placed in a non-transparent tin. The ward personnel participating during the discharge interview performed the randomisation. The personnel blindly drew a marble from the tin. A green marble represented IVR calls every second day, while a red marble indicated IVR calls every fourth day. Once a marble had been drawn, it was considered used and was thrown away. Follow-up calls started the day after discharge and went on for 31 consecutive days or until the first visit in outpatient care, i.e. the participants were contacted 1–15 or 1–7 times in the every second day group and every fourth day group respectively.

### Inclusion and exclusion criteria

Consecutive inpatients treated between December 2008 and November 2009 at the emergency unit, Department of Child & Adolescent psychiatry in Malmö, Sweden were included. Patients younger than thirteen or older than seventeen years of age were excluded from the study. Patients with mental retardation and non-Swedish speaking individuals were also barred. Patients who could not be discharged to outpatient care were also excluded. Patients with their first visit in outpatient care on the day of discharge or the day after; deviantly registered patients; and patients treated less than 24 hours were also barred. If the patients had no access to a personal cell-phone they were offered to borrow one from the unit.

### Procedure

**Figure 1:**
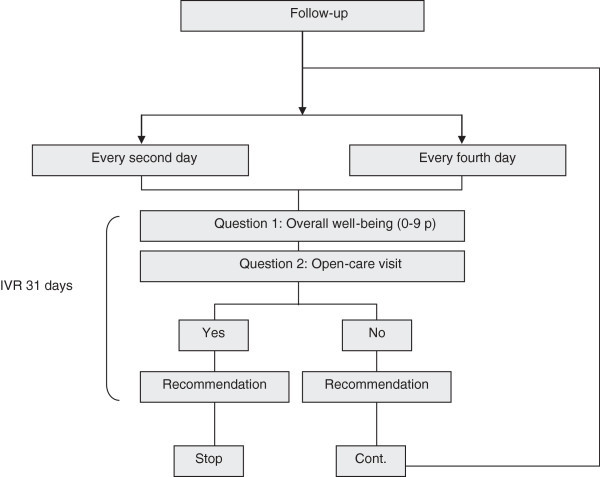
**Flow-chart - interactive voice response (IVR).**

Automated attempts to contact the patients were made each hour between 5 PM and 9 PM, every second and fourth day respectively. All calls, successive or non-successive were logged in an Access-file in the central computer. There was no verification on the patients’ identity. At each call patients were asked by the pre-recorded voice to rate their current mood. They were also asked to respond to whether they had been to an outpatient care consultation since the last time they were contacted by the IVR system. Patients answering that they had not visited an outpatient care unit were called again according to schedule, while patients answering that they had been to an outpatient care consultation after discharge were not called again. At the end of each call, patients were informed to talk to someone trusted or to contact the emergency unit if experiencing accentuated symptoms. At the last call, which occurred 31 days after discharge, or after the first outpatient care visit, the participant was informed that no additional calls were to be made from the IVR system (Figure 1).

### Measures

Sex, age, treatment period, and diagnoses were retrieved from clinical files. Patients rated their overall wellbeing on a 10-point Visual analogue scale (VAS) (Bowen et al. 2004 Mar), where 0 indicated worst possible mood and 9 that patients were feeling as good as possible.

### Statistics

Age was dichotomised into “mid-adolescents” (13–15 years) and “late-adolescents” (16–17 years) groups (Whitbeck et al. 2008; Kovacs et al. 2003), and treatment period was dichotomised into short treatment period (< 3 days) and long treatment period (≥ 3 days) (Case et al. 2007). Diagnoses were clustered into three categories: mood disorders; neurotic, stress-related, and somatoform disorders; and other diagnoses (World Health Organization 2010). Calculations of group differences were performed with Fisher’s exact χ^2^-test. A linear model with random coefficients for subjects (repeated measurements) was used for calculations of differences in mood estimations when answering. Logistic regression was used for calculations of differences in answering frequency. A *p*-value of < 0.05 was considered significant (Altman 1994).

## Results

### Acceptance

**Figure 2:**
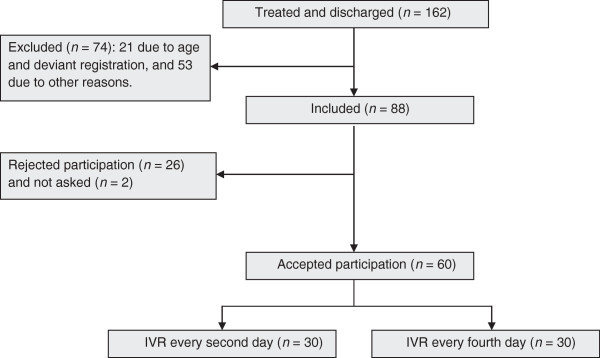
**Schematic representation of included and excluded patients.**

During recruitment 162 patients were treated at the emergency unit. Twenty-one of these patients were excluded due to age (N = 19) or deviant registration (N = 2). A total of fifty-tree patients were excluded from the study due to language difficulties (N = 20); outpatient care visit on the day of or the day after discharge (N = 13); mental retardation (N = 11); need for a prolonged inpatient period (N = 8); and less than 24 hours of inpatient care (N = 1). Amongst 88 possible included individuals, two individuals were not asked if they wanted to contribute. Twenty-six patients declined participation; 23 of these chose not to participate of their own will, while in three cases the parents did not want their adolescents to participate. A total of 60 (68%) patients accepted to participate, and were randomised to be contacted every second day (N = 30) or every fourth day (N = 30) (Figure 2). All 60 patients had their own mobile phone.

### Baseline comparisons

There was no baseline difference concerning sex, age, treatment duration or diagnosis between those excluded due to other reasons than age and deviant registration (N = 53) and included patients (N = 88), nor between the patients who accepted (N = 60) or turned down (N = 26) participation. Neither did we find any baseline differences between patients randomised to be contacted every second (N = 30) or every fourth (N = 30) day (Table 1).

**Table 1:** **Sample characteristics, randomisation groups and level of significance**

Patients	Every second day ***N =*** 30	Every fourth day ***N =*** 30	Level of significance
*Sex*			
Boys	11 (36.5%)	13 (43.5%)	ns
Girls	19 (63.5%)	17 (56.5%)	ns
*Age*			
13-15 years	14 (46.5%)	11 (36.5%)	ns
16-17 years	16 (53.5%)	19 (63.5%)	ns
*Treatment duration*			
< 3 days	14 (46.5%)	15 (50.0%)	ns
≥ 3 days	16 (53.5%)	15 (50.0%)	ns
*Diagnosis, ICD-10*			
Mood disorders, F30-F39	13 (43.0%)	8 (27%)	ns
Neurotic, stress-related and somatoform disorders, F40-F48	9 (30.0%)	11 (36.5%)	ns
Other disorders	8 (27.0%)	11 (36.5%)	ns

### Response rate

Each individual (N = 60) responded on average 91% of their calls. An answering frequency of 100% was obtained by 71% (N = 42). Eighty-eight percent (N = 53) responded to at least 75% of the calls. The mean (SD) number of calls in the follow-up period were 9.7 (5.9) and 3.7 (2.5) times in the 2-day and 4-day group respectively. The probability of answering on at least 75% of the occasions was the same in both randomisation groups. Sex, age, treatment period or different diagnoses did not affect the answering frequency.

### Current mood during follow-up

There were no group differences on current mood during the follow-up period between the randomisation groups. Boys estimated their mood state on average as 3.52 units higher than the girls (CI = 2.65, 4.48, *p* < 0.0001). Patients with an inpatient treatment period with fewer than three days estimated their mood on average as 1.16 units higher than those with a treatment length for at least three days (CI = 0.23, 2.10, *p* = 0.02). The mean (SD) mood state for patients with an inpatient treatment period fewer than three days was, for girls 4.33 (1.71), and for boys 7.31 (1.15). For patients with an inpatient treatment period of at least three days the values were 3.02 (1.76) for girls and 6.86 (1.61) for boys.

### Diagnoses

There were no interactions between different diagnoses and sex, age, treatment period, randomisation groups and response rate.

## Discussion

Our major finding was that automated IVR works as a self-reporting follow-up technique on current mood in adolescents discharged from emergency psychiatric inpatient care. Having a personal cell-phone was a necessary condition for participation. No one refused to participate because they did not have a personal cell-phone. A personal cell-phone reduces the risk that somebody else answers incoming calls. The participants answered on average 91% of their calls. A response rate of 100% was obtained by 71% (N = 42). There were no differences in response rates between adolescents followed up every second or every fourth day, suggesting that a 2-day interval is not necessary in order to achieve a high response rate. Calling every 4- days would conserve resources and response burden. Our response rate was higher than the results in two comparable outpatient studies, where the response rates were 72% and 14% respectively (Kaminer et al. 2006; Blackstone et al. 2009). One explanation could be that the patients in our study were called by the IVR system, while the patients in the two other studies called the IVR system themselves. Another explanation could be that our study suffers from positive selection bias. The acceptance rate in our study was 68%; in the other two studies it was 84% and 73% respectively (Kaminer et al. 2006; Blackstone et al. 2009). However, we found no baseline differences between patients rejecting participation and patients who chose to participate. The response rate in our and Kaminer’s (Kaminer et al. 2006) studies were higher compared to the response rate in Blackstone’s study (Blackstone et al. 2009). One interpretation might be that high response rates require follow-ups at least twice a week. Two other factors that might explain the low response rate in Blackstone’s study could be the extended follow-up period, and that participants probably lived in a more vulnerable social context (Londino et al. 2003; Mason et al. 2009) compared to the patients in our and Kaminer’s studies (Kaminer et al. 2006).

Our second finding concerns current mood during follow-up. We found no differences in reports on current mood between telephone contacts on every second or fourth day. To further evaluate if contact frequency could influence current mood, diagnoses, specific treatment options, and other supportive factors must be taken into consideration (Blackstone et al. 2009).

We also found that the boys estimated their current mood as much better than the girls. An explanation could be a more severe psychiatric picture among the girls, e.g. higher co-morbidity and more severe depressions while in inpatient treatment (Mason et al. 2009). From a gender perspective, girls have been found to report higher levels of distress and are more likely to perceive themselves as having an emotional problem than boys presenting with a similar level of symptoms (World Health Organization 2002).

Finally, we found that patients with a treatment period of fewer than three days displayed a better mood during follow-up compared to patients treated for three days or more. For adolescents in general the major recovery often occurs early in treatment (Green 2002). Our results might be explained by less psychiatric symptoms and a stronger family involvement (Londino et al. 2003; Mason et al. 2009; Green 2002) among patients treated for fewer than three days compared to those patients treated for longer periods.

### Strengths

The present study poses a research question of importance to IVR researchers who have limited empirical evidence to guide their decisions on the appropriate IVR assessment frequency. The 3-week interval between discharge and outpatient follow-up is long, and a lot of clinical change can occur. Monitoring of patients between these events is important in order to provide optimal care.

### Limitations

We did not validate the VAS with a screening questionnaire; however previous studies in psychiatry have shown that VAS can provide valid data on mood (Bowen et al. 2004 Mar). The short survey could explain part of the high response rate, though studies with more extended surveys including 24 items have shown similar response rates (Andersson et al. 2007). Patients were randomised to be followed-up every second or every fourth day. The follow-up frequencies were guided by differences in response rates in frequent (Kaminer et al. 2006) and non-frequent contact (Blackstone et al. 2009) IVR studies. To evaluate IVR treatment effects, it would be necessary to randomise either to a different follow-up technique, e.g. mobile applications, handheld computers, paper- or web surveys or to an untreated control group.

### Future implications

Future research could compare 4-day vs. longer (e.g., 6-day) intervals to identify where the drop-off in response rate occurs. A control group and a longer follow-up period could make it possible to evaluate long-term treatment effects. In clinical settings, real time monitoring can identify adolescents most vulnerable or distressed and link them directly to telephone support from staff at an emergency unit. IVR can save clinician time and monitor the quality of care. Another direction for research is to use the IVR system to provide targeted feedback based on a person’s response to the mood item, e.g. encouraging or congratulating good moods vs. making suggestions for low mood.

## Conclusions

Automated IVR is a feasible technology for collecting self-reported data on current mood among adolescents discharged from emergency psychiatric inpatient care. It seems viable to perform IVR follow-ups a couple of times a week. The technology offers new promising ways for remote collection of self-reported data.

### Ethics

No compensation was offered. Written informed consent was received from both patients and parents. The Regional ethical review board in Lund approved the study 2007-10-23 (Nr 460/2007).
